# Stroke Severity and Outcomes in Patients With Newly Diagnosed Atrial Fibrillation

**DOI:** 10.3389/fneur.2021.666491

**Published:** 2021-06-29

**Authors:** Kotaro Watanabe, Shuhei Okazaki, Takaya Kitano, Shintaro Sugiyama, Mariko Ohara, Hideaki Kanki, Tsutomu Sasaki, Manabu Sakaguchi, Hideki Mochizuki, Kenichi Todo

**Affiliations:** Department of Neurology, Osaka University Graduate School of Medicine, Osaka, Japan

**Keywords:** atrial fibrillation, acute ischemic stroke, outcome, severity, newly diagnosed atrial fibrillation

## Abstract

**Background and Purpose:** Once a stroke occurs in a patient with atrial fibrillation (AF), it is likely to be severe. Patients with newly diagnosed AF after stroke and those with known AF before stroke have different background characteristics, yet the difference in stroke severity has not been sufficiently evaluated. In the current study, we compared the stroke severity and in-hospital outcomes between these patient groups.

**Methods:** We retrospectively analyzed a database of 196 patients with acute ischemic stroke and AF between January 2010 and October 2019. We divided the patients into two groups: patients with “newly diagnosed AF” and those with “known AF.” We assessed the stroke severity using the National Institutes of Health Stroke Scale (NIHSS) score on admission and in-hospital outcomes using the modified Rankin Scale (mRS) score at discharge.

**Results:** The proportion of newly diagnosed AF was 33% (64/196). There were no differences in age, hypertension, diabetes mellitus, and past history of heart failure between patients with newly diagnosed AF and those with known AF. Patients with newly diagnosed AF were associated with a lower proportion of male sex (male; 50 vs. 67%, p < 0.05), a lower proportion of past history of stroke (12 vs. 35%, p < 0.01), a lower CHA_2_DS_2_-VASc score (median [interquartile range]; 3 [2–4] vs. 3.5 [3–5], p < 0.01), and a lower proportion of pre-stroke oral anticoagulation (5 vs. 59%, p < 0.01). There were no differences in the NIHSS score on admission (12 [4–19] vs. 9 [3–19]) or the mRS score at discharge (3 [1–5] vs. 3 [1–5]). After adjustment for relevant covariates, newly diagnosed AF was not associated with the NIHSS score on admission [adjusted common odds ratio (OR), 0.85; 95% confidence interval (CI), 0.45–1.60] or the mRS score at discharge (adjusted common OR, 1.67; 95% CI, 0.88–3.18). After propensity score matching, newly diagnosed AF was not associated with the NIHSS score on admission (adjusted common OR, 0.91; 95% CI, 0.48–1.73) and the mRS score at discharge (adjusted common OR, 1.77; 95% CI, 0.92–3.43).

**Conclusion:** Stroke severity and in-hospital outcomes in patients with newly diagnosed AF did not differ from those in patients with known AF after adjustment for clinically relevant factors. The importance of detection of latent AF and subsequent anticoagulation in preventing severe stroke should be further emphasized.

## Introduction

Ischemic stroke occurring in patients with atrial fibrillation (AF) is likely to be severe or fatal (1, 2). In patients with AF, oral anti-coagulation can substantially reduce the risk of stroke (3, 4). Therefore, detection of AF and subsequent anticoagulation is crucial for stroke prevention. However, half of AF patients are asymptomatic (5). It is difficult to detect AF in such asymptomatic patients. Among acute ischemic stroke patients with AF, 7.8 to 36.2% were diagnosed as having AF for the first time after the index stroke (6–9). Patients with AF newly diagnosed after stroke and those with AF known before stroke have different background characteristics (6–9). However, differences in stroke severity and outcomes have not been sufficiently evaluated. In the current study, we analyzed stroke severity and the in-hospital outcomes in patients with newly diagnosed AF and those with known AF.

## Materials and Methods

### Ethics Statement

This study complied with the Declaration of Helsinki guidelines for investigations involving humans, and all methods were carried out in accordance with relevant guidelines and regulations for observational studies. The studies involving human participants were reviewed and approved by Institutional Review Boards of Osaka University Medical Hospital. Because we used clinical information obtained in routine clinical practice, written informed consent for participation was not required for this study in accordance with the Ethical Guidelines for Medical and Health Research Involving Human Subjects in Japan. No potentially identifiable human images or data are presented in this study.

### Patient Recruitment

We retrospectively analyzed a database of 196 patients with acute ischemic stroke and AF between January 2010 and October 2019. We enrolled consecutive patients who were aged 20 years or older and hospitalized within 7 days of the onset of an acute ischemic stroke to the Department of Neurology and Stroke Center at Osaka University Hospital.

### Variables and Measurements

We obtained clinical information from the hospital charts. We used the following clinical data for the analyses in the current study: age, sex, hypertension, diabetes mellitus, past history of heart failure, past history of stroke, pre-stroke CHA_2_DS_2_-VASc (congestive heart failure, hypertension, age ≥75 years, diabetes mellitus, previous stroke, vascular disease, age 65–74 years, and sex category) score (10), pre-stroke modified Rankin Scale (mRS) score, pre-stroke oral anticoagulation, the National Institutes of Health Stroke Scale (NIHSS) score on admission, intravenous tissue plasminogen activator (IV-rtPA), endovascular therapy, and mRS score at discharge.

To assess the association of newly diagnosed AF with stroke severity and in-hospital outcomes, the study outcome measurements were set as the NIHSS score on admission and the mRS score at discharge.

### Statistical Analyses

We defined “newly diagnosed AF” as AF diagnosed for the first time after the index stroke using a 12-lead electrocardiogram or 24-h Holter electrocardiogram, and “known AF” as AF diagnosed before the index stroke at any facilities. We compared the above clinical information between patients with newly diagnosed AF and those with known AF. Continuous variables were analyzed using the Mann–Whitney *U*-test and expressed as median values and interquartile ranges (IQRs). Categorical data were analyzed using the chi-square test and expressed as numbers and percentages. In addition, we compared the clinical information between patients with NIHSS scores <10 and ≥10 on admission and between patients with mRS scores ≤2 and >2 at discharge.

We developed multivariate logistic regression models to assess the independent association of newly diagnosed AF with stroke severity and outcomes. In model 1, using the ordinal score of NIHSS on admission as an outcome variable, we entered newly diagnosed AF, male sex, past history of stroke, pre-stroke CHA_2_DS_2_-VASc score, pre-stroke mRS score, and pre-stroke oral anticoagulation as predictive variables. In model 2, using the ordinal score of mRS at discharge, we entered newly diagnosed AF, male sex, past history of stroke, pre-stroke CHA_2_DS_2_-VASc score, pre-stroke mRS score, pre-stroke oral anticoagulation, the NIHSS score on admission, IV-rtPA, and endovascular therapy.

In addition, we developed a propensity score-matched cohort. We used a logistic regression model to develop the propensity score. The variables for the propensity score included male sex, past history of stroke, pre-stroke CHA_2_DS_2_-VASc score, pre-stroke mRS score, and pre-stroke oral anticoagulation. Because the number of the patients with newly diagnosed AF was fewer than that with known AF, the patients with known AF were matched to those with newly diagnosed AF using a 1:1 matching technique (11). We developed conditional logistic models with pairs of patients with newly diagnosed AF and those with known AF.

Statistical significance was set at p < 0.05. We conducted all analyses with R software using the “rms” package (version 3.3.3, F Foundation for Statistical Computing, Vienna, Austria) (12).

## Results

### Baseline Characteristics

**Figure 1:**
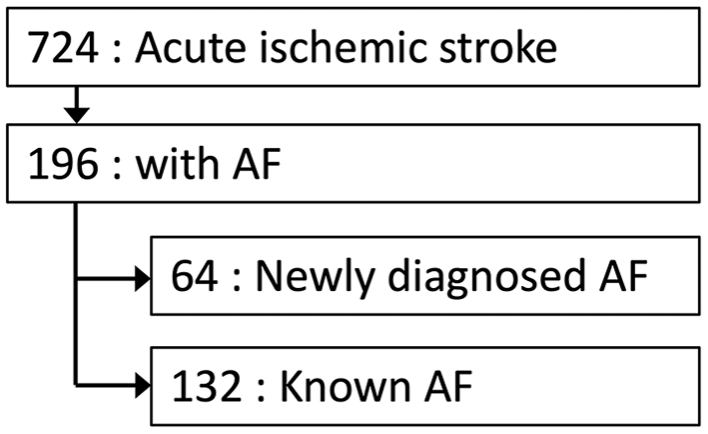
Flow chart for study population. AF indicates atrial fibrillation.

Among the 724 patients with acute ischemic stroke, 196 patients were diagnosed with AF, and the population of newly diagnosed AF was 33% (64/196) (Figure 1). The differences in clinical characteristics between the patients with newly diagnosed AF and those with known AF are shown in Table 1. There were no differences in age, hypertension, diabetes mellitus, and past history of heart failure between the two groups. Patients with newly diagnosed AF were associated with a lower proportion of male sex (50 vs. 67%, p < 0.05), a lower proportion of past history of stroke (12 vs. 35%, p < 0.01), lower pre-stroke CHA_2_DS_2_-VASc scores (median [IQR]; 3 [2–4] vs. 3.5 [3–5], p < 0.01), a lower proportion of pre-stroke mRS score of ≥1 (22 vs. 33%, p < 0.05), and a lower proportion of pre-stroke oral anticoagulation (5 vs. 59%, p < 0.01).

**Table 1:** Clinical characteristics.

	**Newly diagnosed AF (*N* = 64)**	**Known AF (*N* = 132)**	***p* value**
Age, median (IQR), years	76 (67–83)	77 (72–83)	0.40
Male sex, no. (%)	32 (50%)	88 (67%)	<0.05
Hypertension, no. (%)	34 (53%)	82 (62%)	0.23
Diabetes mellitus, no. (%)	8 (12%)	26 (20%)	0.21
Past history of heart failure, no. (%)	2 (3%)	11 (8%)	0.17
Past history of stroke, no. (%)	8 (12%)	46 (35%)	<0.01
Pre-stroke CHA_2_DS_2_-VASc score, median (IQR)	3 (2–4)	3.5 (3–5)	<0.01
Pre-stroke mRS score ≥1, no. (%)	14 (22%)	44 (33%)	<0.05
Pre-stroke oral anticoagulation, no. (%)	3 (5%)	78 (59%)	<0.01
IV-rtPA, no. (%)	18 (28%)	17 (13%)	0.009
Endovascular therapy, no. (%)	15 (23%)	30 (23%)	0.91

*IQR, interquartile range; IV-rtPA, intravenous recombinant tissue plasminogen activator*.

Patients with NIHSS scores <10 on admission were associated with a higher proportion of male sex (75 vs. 49%, p < 0.01), a higher proportion of past history of stroke (36 vs. 20%, p < 0.05), and a higher proportion of pre-stroke oral anticoagulation (49 vs. 34%, p < 0.05) (Supplementary Table 1). Patients with mRS scores ≤2 at discharge were associated with a higher proportion of male sex (74 vs. 52%, p < 0.01), lower pre-stroke CHA_2_DS_2_-VASc scores (3 [2–4] vs. 4 [3–5], p < 0.01) and a lower proportion of pre-stroke mRS score of ≥1 (15 vs. 40%, p < 0.01) (supplementary Table 2).

After the propensity score matching, 64 patients in each group were matched with the counterparts. Patients with newly diagnosed AF were associated with a lower proportion of pre-stroke oral anticoagulation (supplementary Table 3).

### Outcomes

**Figure 2:**
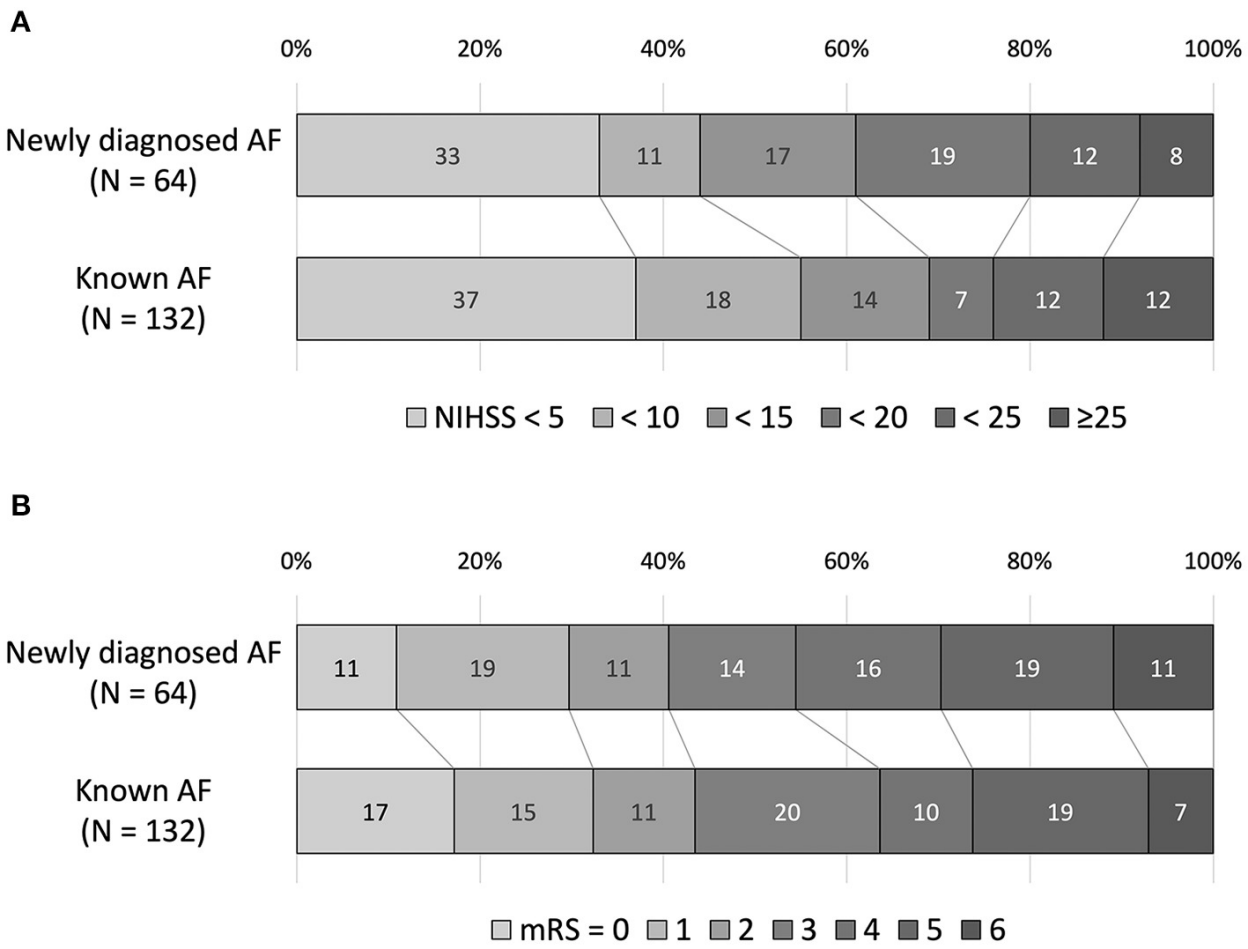
The National Institutes of Health Stroke Scale (NIHSS) score on admission **(A)** and the modified Rankin Scale (mRS) score at discharge **(B)**. There was no difference in the NIHSS score on admission and the mRS score at discharge between the two groups.

There was no difference in stroke severity (NIHSS score on admission; 12 [4–19] in patients with newly diagnosed AF vs. 9 [3–19] in patients with known AF). The distributions of NIHSS scores on admission are shown in Figure 2. After adjustment for clinically relevant factors, newly diagnosed AF was not associated with the NIHSS score on admission [adjusted common odds ratio (OR), 0.85; 95% confidence interval (CI), 0.45–1.60) (Table 2). There was no difference in the in-hospital outcome (mRS score at discharge; 3 [1–5] in patients with newly-diagnosed AF vs. 3 [1–5] in patients with known AF). The distributions of mRS scores at discharge are shown in Figure 2. After adjustment for clinically relevant factors, newly diagnosed AF was not associated with the mRS score at discharge [adjusted common OR, 1.64; 95% CI, (0.86–3.15)] (Table 2).

**Table 2:** Adjusted common odds ratios for outcomes.

**Model**	**Outcome**	**Predictor variable**	**Adjusted common odds ratio (95% confidence interval)**	***p* value**
1	NIHSS score on admission	Newly diagnosed AF	0.85 (0.45–1.60)	0.62
		Male sex	0.52 (0.30–0.92)	<0.05
		Past history of stroke	0.42 (0.20–0.89)	<0.05
		Pre-stroke CHA_2_DS_2_-VASc score, per 1-point increase	1.08 (0.86–1.36)	0.50
		Pre-stroke mRS score, per 1-point increase	1.12 (0.92–1.36)	0.26
		Pre-stroke oral anticoagulation	0.71 (0.38–1.35)	0.30
2	mRS score at discharge	Newly diagnosed AF	1.64 (0.86–3.15)	0.14
		Male sex	1.00 (0.55–1.82)	0.99
		Past history of stroke	0.55 (0.26–1.18)	0.12
		Pre-stroke CHA_2_DS_2_-VASc score, per 1-point increase	1.18 (0.93–1.50)	0.17
		Pre-stroke mRS score, per 1-point increase	1.67 (1.34–2.10)	<0.01
		Pre-stroke oral anticoagulation	1.18 (0.63–2.22)	0.60
		NIHSS score on admission, per 1-point increase	1.24 (1.18–1.29)	<0.01
		IV-rtPA	1.25 (0.57–2.74)	0.57
		Endovascular therapy	0.26 (0.11–0.61)	<0.01

*IV-rtPA, intravenous recombinant tissue plasminogen activator; mRS, modified Rankin Scale; NIHSS, National Institutes of Health Stroke Scale*.

After propensity score matching, newly diagnosed AF was not associated with the NIHSS score on admission (adjusted common OR, 0.91; 95% CI, 0.48–1.73) and the mRS score at discharge (adjusted common OR, 1.77; 95% CI, 0.92–3.43) (supplementary Table 4).

## Discussion

In the current retrospective analysis of a single-center registry, stroke severity and in-hospital outcomes did not differ between patients with newly diagnosed AF and those with known AF. It has already been reported that the NIHSS score did not differ between groups (7). In the present study, we confirmed and further clarified that the result was still the same after adjustment for clinically relevant factors.

The CHA_2_DS_2_-VASc score is originally a clinical risk stratification system commonly used to assess the risk of stroke in patients with AF (10). In addition, a population-based study has reported that higher CHA_2_DS_2_-VASc scores are associated with AF detection (13). This is consistent with our results that the patients with known AF had higher pre-stroke CHA_2_DS_2_-VASc scores than those with newly diagnosed AF. Moreover, previous reports have shown that higher CHA_2_DS_2_-VASc scores are associated with the worse stroke severity and outcomes (14–16). In our cohort, higher CHA_2_DS_2_-VASc scores were associated with a worse in-hospital outcome.

Male patients have been shown to have a higher incidence of AF than female patients (17). This is consistent with our findings of a higher proportion of male sex in patients with known AF. Among ischemic stroke patients with AF, female patients have been reported to be associated with a worse stroke outcome (18). In our cohort, female patients were associated with more severe strokes on admission and worse in-hospital outcomes.

Guidelines recommend oral anti-coagulant therapy for stroke prevention in patients with AF who are at high risk for stroke (10). However, among patients with acute ischemic stroke and known AF, only 30 to 39% were receiving oral anticoagulant therapy prior to the index stroke (19–22). These studies have reported that oral anticoagulant therapy for patients with AF may reduce stroke severity and improve the outcome of a stroke (19–22). In our cohort, pre-stroke oral anticoagulation was associated with a lower NIHSS score on admission.

Although these background characteristics relating to newly diagnosed AF influenced stroke severity or outcomes, the current study did not show differences in stroke severity or the in-hospital outcomes between patients with newly diagnosed AF and those with known AF. It is presumed that the effect of the lower CHA_2_DS_2_-VASc scores and lower pre-stroke mRS scores on stroke severity and the in-hospital outcomes in patients with newly diagnosed AF may have offset the effect of a lower proportion of male gender and lower proportion of patients with anticoagulation. Even after the adjustment of difference in background factors using propensity score matching, there was no difference in stroke severity and in-hospital outcomes between patients with newly diagnosed AF and those with known AF.

We did not explore all of the potential factors that may differ between patients with newly diagnosed AF and those with known AF. A previous study showed that patients with newly diagnosed AF have a low proportion of underlying cardiac disease (7), while another study showed that patients with both newly diagnosed and known AF share similar cardiovascular risk profiles and echocardiographic findings (6). Instead of echocardiographic findings, we assessed the past history of heart failure. Insular infarctions have also been reported to be associated with newly diagnosed AF (6). In contrast, another study showed that insular infarction did not predict newly diagnosed AF (23). The association between insular infarction and newly diagnosed AF is still under debate.

Several limitations of this study should be considered. First, this was a retrospective observational study of a small cohort at a single center. Thus, larger cohort studies and validation studies are needed. Second, given the retrospective design, a lack of standardized work-up for AF detection may lead to potential selection bias in patients with newly diagnosed AF. In addition, the diagnosis of AF was made by the attending physician. This may also lead to potential selection bias. Third, known AF was defined based on medical documentation, which may also lead to potential selection bias. Fourth, this was a confirmatory study of a previous report; however, we could show the results in more detail.

## Conclusion

We revealed that stroke severity and in-hospital outcomes in patients with newly diagnosed AF did not differ from those in patients with known AF. Given the stroke severity in patients with AF, it should be more emphasized that detection of latent AF and subsequent anti-coagulation is crucial for prevention of severe stroke.

## Data Availability Statement

The raw data supporting the conclusions of this article will be made available by the authors, without undue reservation.

## Ethics Statement

The studies involving human participants were reviewed and approved by Institutional Review Boards of Osaka University Medical Hospital. Written informed consent for participation was not required for this study in accordance with the national legislation and the institutional requirements.

## Author Contributions

KW, SO, and KT designed and conceptualized the study and analyzed the data and drafted the manuscript for intellectual content. KW, KT, SS, MO, and MS had major roles in the acquisition of data. TK, HK, TS, MS, and HM revised the manuscript for intellectual content.

## Conflict of Interest

KT serves as a speaker for Pfizer, Bristol-Myers Squibb, Daiichi-Sankyo, and Bayer. The remaining authors declare that the research was conducted in the absence of any commercial or financial relationships that could be construed as a potential conflict of interest.
