# Supplementary material for: Stroke Severity and Outcomes in Patients With Newly Diagnosed Atrial Fibrillation
Source: Front Neurol. 2021 Jun 29;12:666491. doi: 10.3389/fneur.2021.666491 (PMC8275857; doi:10.3389/fneur.2021.666491)
Supplement: Supplementary file 1 [file Table_1.PDF]

Supplementary Material

1 Supplementary Tables

Supplemental Table I: Clinical characteristics according to the NIHSS score on admission

	**NIHSS score < 10 (N = 95)**	**NIHSS score ≥ 10 (N = 101)**	***P value***
Male sex, No. (%)	71 (75%)	49 (49%)	< 0.01
Past history of stroke, No. (%)	34 (36%)	20 (20%)	< 0.05
Pre-stroke CHA_2DS_2-VASc score, median (IQR)	3 (2–5)	3 (2–4)	0.94
Pre-stroke mRS score ≥ 1, median (IQR)	29 (31%)	29 (29%)	0.78
Pre-stroke oral anticoagulation, No. (%)	47 (49%)	34 (34%)	< 0.05

IQR indicates interquartile range; and NIHSS, National Institute of Health Stroke Scale.

Supplemental Table II: Clinical characteristics according to the mRS score at discharge

	**mRS score ≤ 2** **(N = 84)**	**mRS score > 2** **(N = 112)**	***P value***
Male sex, No. (%)	62 (74%)	58 (52%)	< 0.01
Past history of stroke, No. (%)	25 (30%)	29 (26%)	0.55
Pre-stroke CHA_2DS_2-VASc score, median (IQR)	3 (2–4)	4 (3–5)	< 0.01
Pre-stroke mRS score ≥ 1, median (IQR)	13 (15%)	45 (40%)	< 0.01
Pre-stroke oral anticoagulation, No. (%)	34 (40%)	47 (42%)	0.83

IQR indicates interquartile range; and mRS, modified Rankin Scale.

Supplemental Table III: Clinical characteristics of Propensity Score-Matched Cohort

	**Newly diagnosed AF (N = 64)**	**Known AF (N = 64)**	***P value***
Age, median (IQR), y	76 (67–83)	77 (69–81)	0.91
Male sex, No. (%)	32 (50%)	41 (64%)	0.11
Hypertension, No. (%)	34 (53%)	34 (53%)	1.00
Diabetes mellitus, No. (%)	8 (12%)	10 (16%)	0.61
Past history of heart failure, No. (%)	2 (3%)	6 (9%)	0.14
Past history of stroke, No. (%)	8 (12%)	11 (17%)	0.46
Pre-stroke CHA_2DS_2-VASc score, median (IQR)	3 (2–4)	3 (2–4)	0.62
Pre-stroke mRS score ≥ 1, No. (%)	14 (22%)	17 (27%)	0.54
Pre-stroke oral anticoagulation, No. (%)	3 (5%)	10 (16%)	< 0.05
IV-rtPA, No. (%)	18 (28%)	13 (20%)	0.30
Endovascular therapy, No. (%)	15 (23%)	16 (25%)	0.83

IQR indicates interquartile range.

Supplemental Table IV: Adjusted common odds ratios for outcomes of Propensity Score-Matched Cohort

Model	Outcome	Predictor variable	Adjusted common odds ratio (95% confidence interval)	*P value*
1	NIHSS score on admission	Newly diagnosed AF	0.91 (0.48–1.73)	0.77
		Male sex	0.67 (0.32–1.38)	0.27
		Past history of stroke	0.40 (0.13–1.19)	0.10
		Pre-stroke CHA_2DS_2-VASc score, per 1 point increase	1.21 (0.84–1.49)	0.43
		Pre-stroke mRS score, per 1 point increase	1.07 (0.81–1.40)	0.64
		Pre-stroke oral anticoagulation	0.53 (0.18–1.63)	0.27
2	mRS score at discharge	Newly diagnosed AF	1.77 (0.92–3.43)	0.09
		Male sex	0.79 (0.37–1.67)	0.53
		Past history of stroke	1.25 (0.43–3.62)	0.68
		Pre-stroke CHA_2DS_2-VASc score, per 1 point increase	1.16 (0.86–1.56)	0.34
		Pre-stroke mRS score, per 1 point increase	1.40 (1.04–1.90)	< 0.05
		Pre-stroke oral anticoagulation	0.62 (0.20–1.92)	0.41
		NIHSS score on admission, per 1 point increase	1.25 (1.18–1.32)	< 0.01
		IV-rtPA	1.12 (0.47–2.67)	0.80
		Endovascular therapy	0.24 (0.09–0.63)	< 0.01

IV-rtPA indicates intravenous recombinant tissue plasminogen activator; mRS, modified Rankin Scale; and NIHSS, National Institute of Health Stroke Scale.
